# *Thlaspi arvense* attenuates colitis-associated colorectal tumorigenesis through suppression of neutrophil recruitment via the NOD/NF-κB pathway

**DOI:** 10.1186/s13020-026-01381-5

**Published:** 2026-04-17

**Authors:** Ziwei Wang, Wenkai Wang, Chaowei Wang, Bijin Dong, Yunchuan Sun, Xinying He, Ling Bi, Yan Wang

**Affiliations:** 1 The Second Clinical Medicine College, Guizhou University of Traditional Chinese Medicine, Guiyang, 550003 Guizhou China; 2 Department of Medical Oncology, Shuguang Hospital, Shanghai University of Traditional Chinese Medicine, Shanghai, 201203 China; 3 School of Life Science and Technology, ShanghaiTech University, Shanghai, 201210 China; 4 Oncology Department of Cangzhou Hospital of Integrated TCM-WM, Cangzhou, 061000 Hebei China; 5 Chinese Medicine Guangdong Laboratory, Guangzhou, 510045 Guangdong China

**Keywords:** *Thlaspi arvense*, Colitis-associated colorectal cancer, Inflammation-Cancer Transition, Neutrophils, CXCL1/2

## Abstract

**Background:**

In colitis-associated colorectal cancer (CAC), chronic inflammation is the primary driver of tumorigenesis. A critical event in this process is the massive recruitment of neutrophils, which, while part of the host defense, can paradoxically fuel cancer progression. Excessive neutrophil infiltration contributes to sustained mucosal damage through the release of pro-inflammatory mediators and shapes a tumor-promoting microenvironment. Despite their recognized role, therapeutic strategies specifically targeting pathogenic neutrophil recruitment in CAC are limited. *Thlaspi arvense* (TA), a traditional medicinal plant, possesses purported anti-inflammatory properties, suggesting its potential utility against CAC. Therefore, this study was designed to evaluate the efficacy of TA in preventing CAC and to delineate its mechanism of action, particularly its impact on neutrophil-driven inflammation.

**Methods:**

An azoxymethane/dextran sulfate sodium (AOM/DSS) mouse model of CAC was employed. Mice were administered with TA to evaluate its effects on disease severity, as gauged by body weight change, colon length, tumor burden, and survival rate. Histological and immunofluorescence staining were performed to assess mucosal integrity and the expression of tight junction proteins (ZO-1, Claudin-1). Neutrophil infiltration was quantified by flow cytometry and immunofluorescence. The levels of pro-inflammatory cytokines (IL-6, TNF-α, IL-1β, IFN-γ) and neutrophil chemoattractants (CXCL1/2) were measured using ELISA and qPCR, respectively. To specifically probe the CXCL1/2-CXCR2 axis, a CXCR2 inhibitor was used as an interventional control. Mechanistic insights were gained through RNA sequencing and Western blot analysis. Furthermore, molecular docking was performed to predict the binding affinity of key TA constituents to core proteins within the NOD/NF-κB pathway.

**Results:**

The AOM/DSS mouse model successfully recapitulated the hallmark features of CAC. Treatment with TA, especially at higher doses, markedly attenuated the pathological manifestations, including body weight loss, colon shortening, adenoma formation, and severe inflammatory responses. Consistently, the TA administration restored mucosal integrity and significantly upregulated the expression of tight junction proteins ZO-1 and Claudin-1. At a functional level, TA significantly reduced the production of the neutrophil chemoattractants CXCL1 and CXCL2, which and consequently impaired the interaction with CXCR2 on neutrophils and led to a substantial decrease in neutrophil recruitment. Mechanistic investigation further demonstrated that TA exerted its effects by suppressing the activation of key proteins within the NOD/NF-κB signaling pathway. This suppression resulted in diminished CXCL1/2 expression and a consequent attenuation of the neutrophil-driven pro-tumorigenic microenvironment.

**Conclusions:**

We conclude that TA attenuates colitis-associated carcinogenesis by inhibiting the NOD/NF-κB pathway and its downstream CXCL1/2-CXCR2-mediated neutrophil recruitment. This study underscores the value of targeting neutrophil-driven inflammation and positions TA as a viable natural therapeutic for preventing CAC progression.

**Graphical Abstract:**

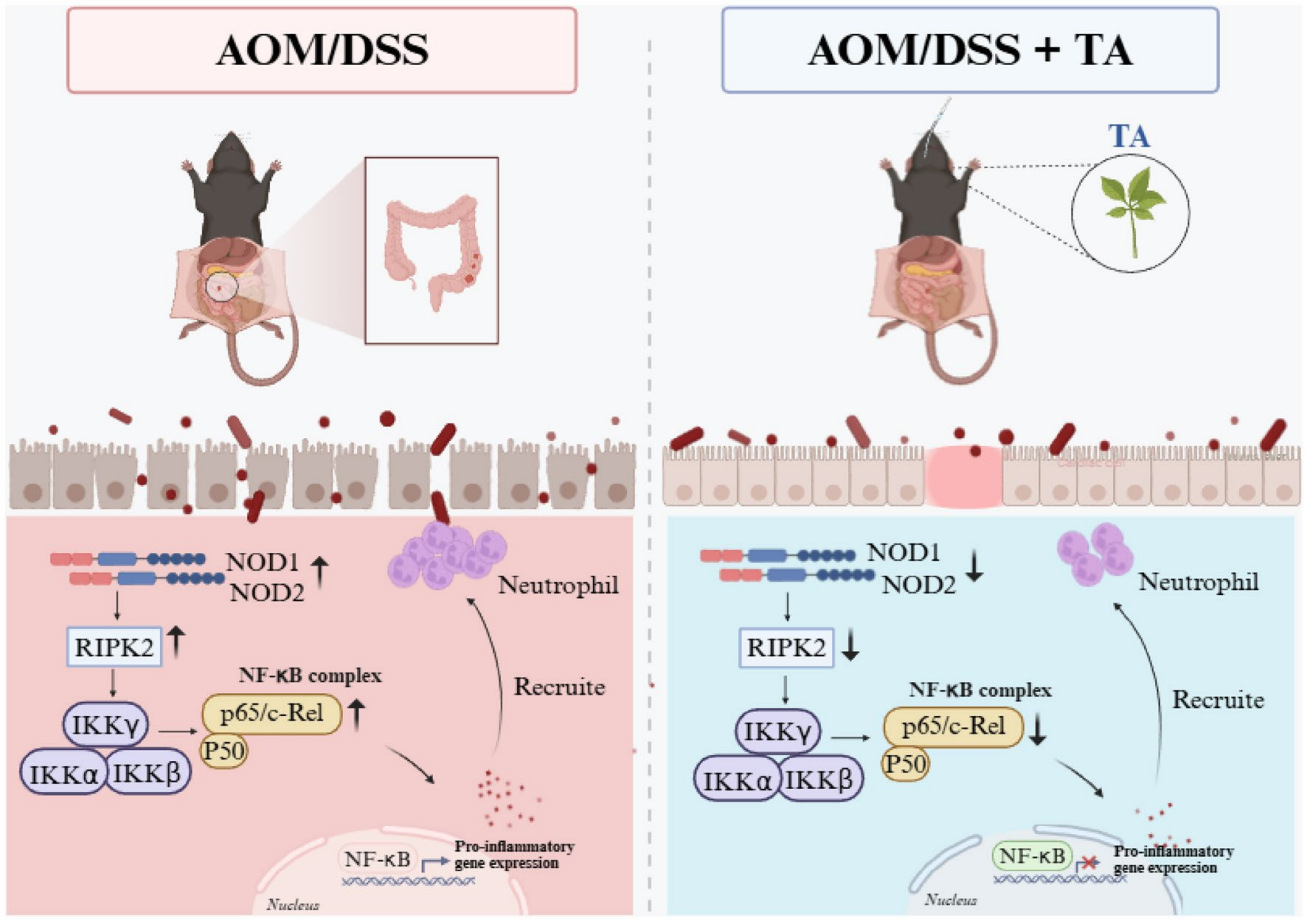

**Supplementary Information:**

The online version contains supplementary material available at 10.1186/s13020-026-01381-5.

## Introduction

Colorectal cancer (CRC) remains a leading cause of cancer-related mortality worldwide [1, 2]. A significant risk factor for CRC is chronic inflammation, particularly in patients with inflammatory bowel disease (IBD), who face a substantially higher risk of developing colitis-associated colorectal cancer (CAC) [3–5]. CAC is an aggressive subtype characterized by accelerated progression and poor prognosis [5]. The management of IBD, aimed at preventing such complications, often involves long-term use of aminosalicylates, corticosteroids, and immunosuppressants [6–8]. However, the efficacy of these therapies is frequently limited by significant side effects, drug resistance, and disease recurrence [9], underscoring the urgent need for safer and more effective treatment strategies.

Within the context of chronic inflammation, neutrophil paradoxical role. As key effector cells of the innate immune system, their regulated activity is essential for host defense [10, 11]. Yet, excessive and sustained neutrophil infiltration becomes a driver of pathology [12, 13]. In the inflamed colon, aberrant neutrophil activation perpetuates mucosal damage, disrupts the epithelial barrier, and creates a pro-tumorigenic microenvironment rich in pro-inflammatory mediators and genotoxic substances [14]. This process not only promotes uncontrolled epithelial proliferation but also facilitates genetic instability, thereby forging a critical link between chronic inflammation and carcinogenesis [15, 16]. Consequently, targeting pathogenic neutrophil recruitment has emerged as a promising therapeutic avenue for intercepting CAC development.

*Thlaspi arvense* (TA) is a medicinal plant recognized as a primary component of the traditional formula Yiyi Fuzi Baijiang San (YYFZBJS), which is used for gastrointestinal disorders and has shown anti-cancer properties [12, 17]. Our previous studies have demonstrated that TA can ameliorate ulcerative colitis through modulation of the gut microbiota and suppression of inflammatory responses [18], and it has shown efficacy against DSS-induced intestinal injury. While these findings highlight TA's multi-target pharmacological potential, its specific impact on the immune dysregulation, particularly on neutrophil-driven inflammation, in the context of CAC remains unexplored.

Therefore, based on the critical role of neutrophils in CAC and the anti-inflammatory properties of TA, we hypothesized that TA might attenuate CAC progression by suppressing neutrophil recruitment. The present study aimed to investigate the protective effects of TA against CAC in a mouse model, with a specific focus on elucidating its role in modulating the NOD/NF-κB signaling pathway and the subsequent CXCL1/2-CXCR2 axis responsible for neutrophil infiltration. Our findings provide novel insights into the mechanism of TA and support its potential as a natural therapeutic agent for CAC.

## Materials and methods

### Reagents and chemicals

Dextran sulfate sodium (DSS, 160,110) was obtained from MP Biomedicals (California, USA).Azoxymethane (AOM, A5486) was obtained from Merck KGaA (Darmstadt, Germany), 5-Aminosalicylic acid (5-ASA, 231,018) was supplied by Shanghai Aide Pharmaceutical Co., Ltd. (Shanghai, China). Sinigrin potassium salt (RFS-H05311805017), isovitexin (RFS-Y11602204022), orientin (RFS-H04411803007), apigenin (RFS-Q00211801029), and luteolin (RFS-M02501909016) were obtained from Refiney Biotechnology Co., Ltd (Chengdu, China). RIPK2 kinase inhibitor (GSK583, 136,57-00-9) were obtained from MCE (Shanghai, US) and Muramyl dipeptide (MDP, 53,678-77-6) were obtained from InvivoChem (Guangzhou, China).

Enzyme-linked immunosorbent assay (ELISA) kits for mouse Interleukin-1 beta (IL‐1β), Interleukin-6 (IL‐6), Tumor Necrosis Factor-alpha (TNF‐α), Interferon-gamma (IFN-γ) and C-X-C Motif Chemokine Ligand 1 (CXCL1) were obtained from Jianglai Biolog (Shanghai, China), and the mouse macrophage inflammatory protein-2 (MIP-2) ELISA kit was purchased from Proteintech (Chicago, USA).

Primary antibodies against the following targets were used for Western blot and immunofluorescence: GAPDH (2118S), NF-κB p65 (8242S), Phospho-NF-κB p65 (Ser536, 3033S), IκBα (4812S), phospho-IκBα (Ser32, 5209S), Ki-67 (34330SF), and Ly-6G (1A8) rat mAb (88876S) from Cell Signaling Technology (Massachusetts, USA); ZO-1 (ab307799), claudin-1 (ab307692), CXCL2 (ab317569) and anti-CD11b (ab184308) from Abcam (Cambridge, UK); NOD1 (DF6378) and NOD2 (DF12125) from Affinity Biosciences (Jiangsu, China); CXCL1 (12,335–1-AP) from Proteintech (Chicago, USA).

For flow cytometry, antibodies were acquired from BD Pharmingen (New Jersey, USA): APC-Cy7 rat anti-mouse CD45 (557,659), FITC rat anti-mouse CD11b (557,396), PE rat anti-mouse Ly-6G (551,461), along with the viability dye7-AAD (559,925).

### Preparation, extraction and component identification of TA

1 kg of TA was soaked in 8 L of purified water for 1 h, followed by decoction using an electric ceramic kettle. The mixture was heated to boiling and maintained at a constant boil for 30 min. The decoction was then filtered through eight layers of gauze to obtain filtrate A. The extraction procedure was repeated using 6 L of purified water to obtain filtrate B. Filtrates A and B were combined and concentrated by rotary evaporation at 60 °C. The resulting concentrate was lyophilized to obtain a freeze-dried powder, with a final yield of 15.34% (w/w). The TA extract was stored in a cool, dry place and protected from light until use. The chemical composition of the TA extract was analyzed by UPLC-MS/MS as previously described [18]. The major bioactive constituents were identified, including Sinigrin potassium salt, isovitexin, orientin, apigenin, and luteolin (Supplementary table 1).

### Animal model and experimental design

Female C57BL/6 J mice (8 weeks of age, 18 ± 2 g) were purchased from the Shanghai SLAC Laboratory Animal Co., Ltd. (SCXK2021-0006) and housed under specific pathogen-free (SPF) conditions with a 12-h light/dark cycle, controlled temperature (22–25 °C), and humidity (around 50%). All procedures were approved by the Institutional Animal Care and Use Committee of Shanghai University of Traditional Chinese Medicine (PZSHUTCM2310190008). After a 7-day acclimation period, mice were used in three independent experiments as described below.

#### Induction of the CAC model

**Fig. 1:**
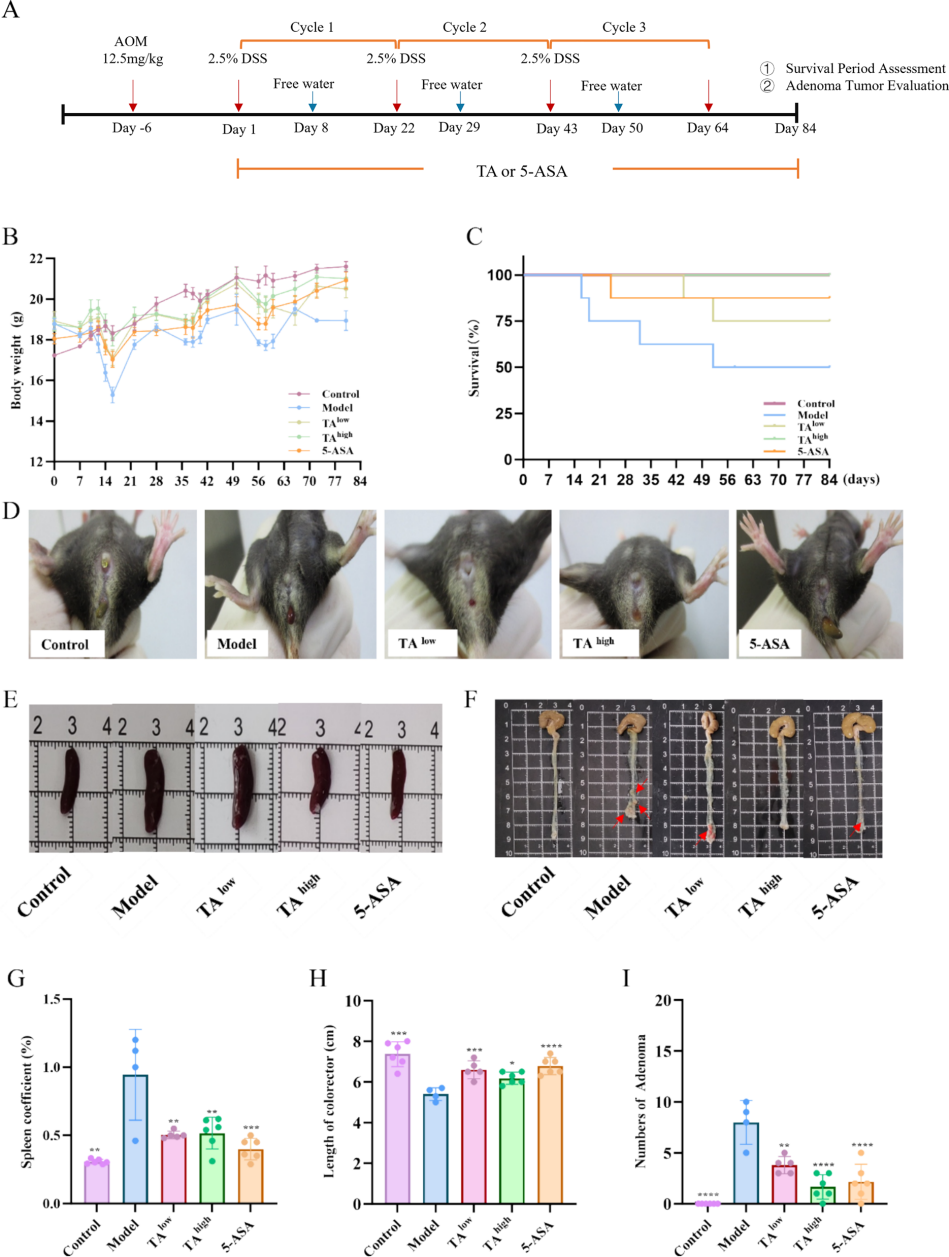
TA attenuates AOM/DSS-induced colorectal tumorigenesis in mice. **A** Experimental workflow of TA intervention in the AOM/DSS-induced colorectal cancer model. The experiments were conducted in two independent cohorts. The first cohort was used to monitor body weight changes and survival outcomes, as shown in (**B**) and (**C**) (n = 8). The second cohort was used to evaluate the effects of TA on colorectal tumor development (n = 6), including macroscopic anal changes (**D**), representative spleen images (**E**), representative colorectal tissue images (**F**), spleen index, intestinal length, and adenoma number (**G**–**I**). *P < 0.05, **P < 0.01, ***P < 0.001, ***P < *0.0001*

On day 0, all mice except those in the control groups received a single intraperitoneal (i.p.) injection of AOM (12.5 mg/kg). One week later, the first DSS cycle was initiated; the second and third DSS cycles were then conducted sequentially. Each cycle consisted of 7 days of 2.5% (w/v) DSS in drinking water followed by 14 days of regular water for recovery, resulting in a total of three DSS cycles [19] (Fig. 1A). Three independent cohorts of mice were used for survival analysis, assessment of colitis severity and tumor burden, and mechanistic studies, respectively mice in each experiment were separately randomized to the indicated treatment groups at the beginning of the study. Body weight and stool consistency were monitored regularly throughout the study, and all treatments were administered daily. At the study endpoint, all mice were euthanized for subsequent analyses.

#### Survival and body weight observation

Mice were randomly divided into five groups (n = 8 per group) to monitor changes in body weight and survival in the AOM/DSS-induced colitis-associated cancer (CAC) model. The experimental groups were as follows (Fig. 1A):

- 1) Control group: Received normal drinking water, daily oral gavage of sterile water daily.
- 2) Model (AOM/DSS) group: Administered sterile water (p.o.) daily.
- 3) TA-Low group: Treated with low-dose TA (2.3 g/kg/day, p.o.) daily.
- 4) TA-High group: Treated with high-dose TA (4.6 g/kg/day, p.o.) daily.
- 5) 5-ASA group: Treated with 5-aminosalicylic acid (200 mg/kg/day, p.o.) daily as a positive control.

#### Efficacy of TA on AOM/DSS-induced colitis

A separate cohort of mice was randomly assigned into five groups (n = 6 per group) to evaluate the effect of TA on ameliorating colitis. The grouping and treatments were identical to those described above (Fig. 1A).

#### Effect of TA on neutrophil-related mechanism

To investigate the role of TA in neutrophil regulation, an additional set of mice was randomized into four groups (n = 5 per group) (Fig. 6A):

- 1) Control group: Normal drinking water, sterile water (p.o.) daily.
- 2) Model (AOM/DSS) group: Treated with the sterile water (p.o.) daily.
- 3) TA group: Treated with the TA (4.6 g/kg/day, p.o.) daily.
- 4) SB225002 group: Treated with the CXCR2 antagonist SB225002 (10 mg/kg/day, i.p.) daily as an interventional control.

### Assessment of disease severity and tissue collection

#### Body weight and survival monitoring

The body weight of the mice was measured and recorded every two to three days throughout the experimental period. The survival status and clinical signs (such as diarrhea and rectal bleeding) of the mice were monitored daily. The survival rate for each group was calculated and plotted.

#### Macroscopic evaluation of colon and spleen

At the endpoint, the spleen was excised, rinsed with PBS, blotted dry, and weighed. The spleen index was calculated as follows: Spleen Index = (spleen net weight / mouse body weight) × 100%. The entire colon was dissected from the cecum to the anus. After carefully removing the mesentery, the colon length was measured on a ruler-lined surface.

### Biochemical and molecular analyses

#### Enzyme-linked immunosorbent assay

The levels of inflammatory cytokines (IL-1β, IL-6, TNF-α) in colon tissue homogenates were quantified using commercial ELISA kits, following the manufacturers' instructions. The absorbance was measured at 450 nm using a microplate reader. The total protein concentration of each homogenate was determined using a BCA protein assay kit for normalization.

#### RNA extraction and quantitative real-time PCR (RT-qPCR)

Total RNA was extracted from intestinal tissues using Trizol reagent (Vazyme, China). RNA concentration and purity were assessed with synthesized using a reverse transcription kit. RT-qPCR was performed using SYBR Green Master Mix on a real-time PCR system. β-actin served as the internal control, and relative mRNA expression levels were calculated using the 2^−ΔΔCt^ method. The primer sequences were as follows:

CXCL1: Forward 5'-CAAACCGAAGTCATAGCCACAC-3', Reverse 5'-TCCGTTACTTGGGGACACCT-3'.

CXCL2: Forward 5'-CCAGACAGAAGTCATAGCCACTC-3', Reverse 5'-TCTTCCGTTGAGGGACAGCA-3'.

#### Western blot analysis

Colon tissues were lysed in RIPA buffer containing protease and phosphatase inhibitors. The total protein concentration was determined using a BCA kit. Proteins were separated by SDS-PAGE, transferred to PVDF membranes, and blocked with 5% non-fat milk or 5% BSA. The membranes were incubated overnight at 4 °C with primary antibodies, followed by incubation with an HRP-conjugated secondary antibody. Protein bands were visualized using a chemiluminescent substrate kit.

### Histological and immunohistochemical analyses

#### Hematoxylin and eosin (H&E) staining

Colon tissues were fixed in 4% paraformaldehyde, embedded in paraffin, and sectioned. After deparaffinization and rehydration, the sections were stained with H&E according to standard protocols. Histopathological features were examined using an automated slide scanner.

#### Immunohistochemistry (IHC)

Deparaffinized sections underwent antigen retrieval in citrate buffer. After blocking with 5% BSA, sections were incubated overnight at 4 °C with primary antibodies. Subsequently, sections were incubated with an HRP-conjugated secondary antibody, and signals were developed using a DAB substrate. The sections were counterstained with hematoxylin and imaged under a light microscope.

#### Immunofluorescence (IF) staining

For IF, deparaffinized sections were subjected to antigen retrieval and blocked with 3% BSA. Sections were incubated sequentially with primary antibodies against ZO-1 and Claudin-1, followed by their respective fluorescently labeled secondary antibodies (Alexa Fluor 488 and CY3). Nuclei were stained with DAPI. Images were captured using a digital slide scanner.

### Flow cytometry

Colorectal tissues were minced and digested in collagenase solutions. The resulting cell suspension was filtered through a 70 μm strainer. After Fc receptor blocking, cells were stained with fluorescently conjugated antibodies against CD45, CD11b, and Ly-6G, along with the viability dye 7-AAD. Neutrophils (CD45^+^CD11b^+^Ly-6G^+^) were identified and analyzed using a flow cytometer.

### RNA Sequencing Analysis

Total RNA was extracted from colon tissues, and RNA quality was verified. RNA-seq libraries were prepared and sequenced on an Illumina platform. Differential gene expression analysis was performed with criteria of FDR < 0.05 and |fold change|≥ 1.5. Gene set enrichment analysis (GSEA) was conducted to identify significantly enriched signaling pathways.

### Molecular docking validation

The three-dimensional structures of target proteins (NOD1, NOD2, RIPK2) were obtained from the PDB. The 2D structures of the main active components of TA were downloaded from PubChem. Molecular docking was performed using AutoDock Tools and Quick Vina 2.0 to predict binding modes and affinities. Results were visualized with Discovery Studio.

### Cell culture and drug treatment

Human colorectal adenocarcinoma Caco-2 cells were obtained from ATCC and cultured in Dulbecco’s modified Eagle’s medium (DMEM) supplemented with 10% fetal bovine serum (FBS), 1% penicillin–streptomycin, and maintained at 37 °C in a humidified incubator with 5% CO₂. Cells were routinely passaged every 2–3 days and used for experiments at 70–80% confluence. For drug intervention experiments, Caco-2 cells were seeded in appropriate culture plates and allowed to adhere overnight. Cells were pretreated with TA extract at the indicated concentrations or the RIPK2 inhibitor GSK583 (1 μM) for 1 h, followed by stimulation with muramyl dipeptide (MDP; 10 μg/mL) to activate the NOD2 signaling pathway [20, 21]. Control cells received an equivalent volume of vehicle. The final concentration of DMSO did not exceed 0.1% (v/v). After treatment for the indicated time periods, cells or culture supernatants were collected for subsequent analyses, including protein expression, gene expression, and inflammatory cytokine measurements.

### Data analysis

Data are expressed as mean ± SD. GraphPad Prism (v10.1.2) was used for all statistical analyses and graphing. Normality and homogeneity of variance were verified using the Shapiro–Wilk test and Brown-Forsythe test, respectively. For comparisons between the two groups (Control *vs.* Model), an unpaired two-tailed Student’s t-test was used. For comparisons across more than two groups, one-way ANOVA was employed, followed by Tukey’s post-hoc test for multiple comparisons. If the data violated the assumptions of parametric tests, the non-parametric Mann–Whitney U test (for two groups) or Kruskal–Wallis test with Dunn’s post-hoc test (for multiple groups) was used. *P* value < 0.05 was defined as the threshold for statistical significance.

## Results

### TA attenuates AOM/DSS-induced colorectal tumorigenesis in mice

To evaluate the therapeutic potential of TA against CAC, we employed the well-established AOM/DSS mouse model. Compared to the healthy controls, mice in the model group exhibited significant body weight loss after each cycle of DSS challenge, accompanied characteristic symptoms of colitis including diarrhea and hematochezia-common symptoms of colitis [22] (Fig. 1D). By the end of the experiment, the model group survival rate plummeted to 50% (4/8) (Fig. 1B). Notably, interventions with 5-ASA (200 mg/kg/day) or TA at both low (2.3 g/kg/day) and high (4.6 g/kg/day) doses effectively ameliorated the weight loss and significantly enhanced the survival rate, with no hepatotoxicity observed (Fig. 1B, C and Supplementary Fig. 2). Upon dissection, the model group displayed pronounced colon shortening, a hallmark of severe colitis, which was significantly alleviated by both TA and 5-ASA treatments. Furthermore, the administration markedly reduced both the number of colonic adenomas and the spleen index compared to the model group (Fig. 1E-I and Supplementary Fig. 3). These results demonstrate that TA effectively mitigates the development and severity of CAC in mice.

### TA preserves intestinal barrier integrity

**Fig. 2:**
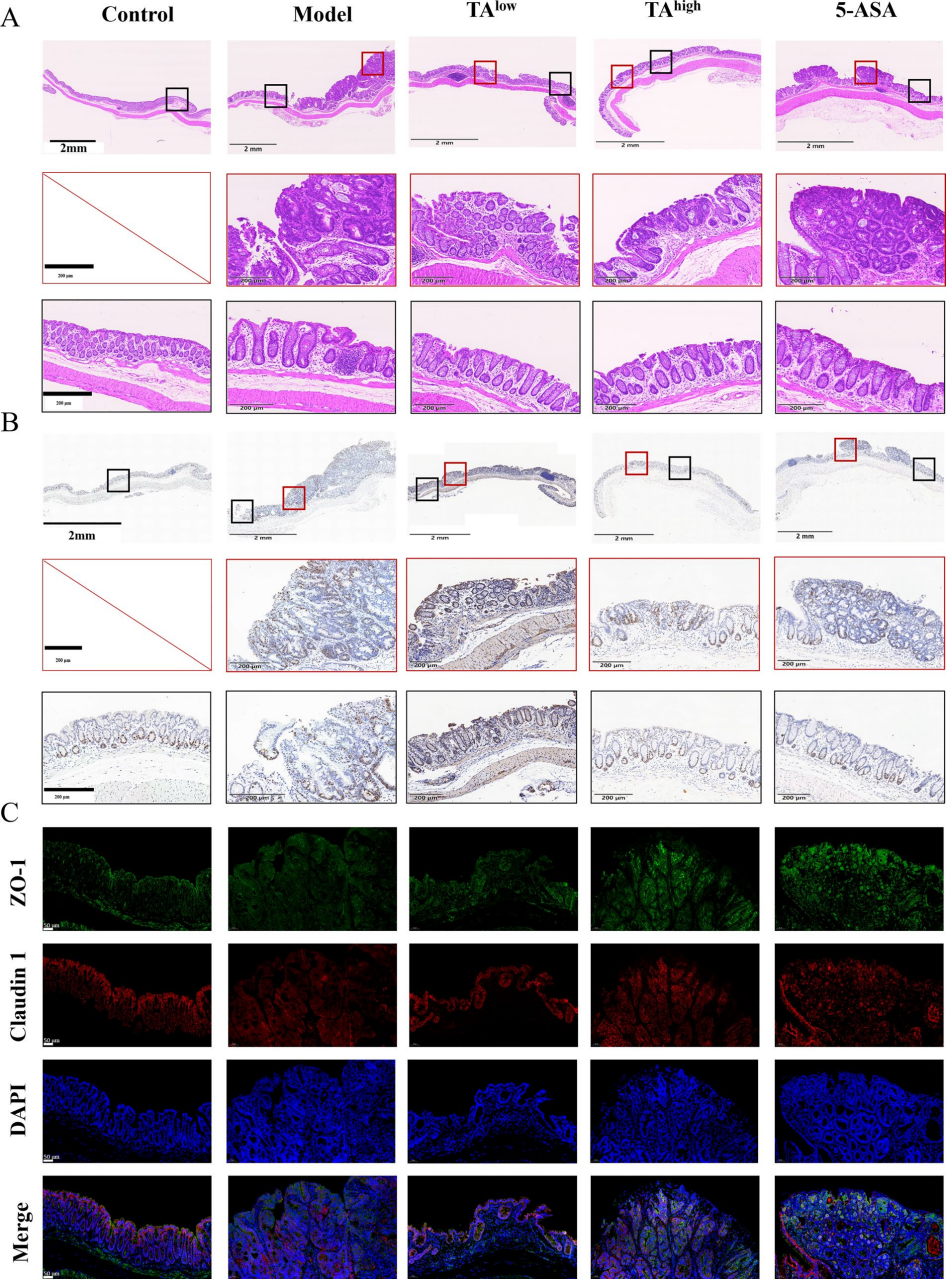
TA preserves intestinal barrier integrity in AOM/DSS-treated mice. **A** Representative hematoxylin and eosin (H&E) staining images of colon tissues, showing histopathological changes under different treatments (10 × magnification). **B** Representative immunohistochemical (IHC) staining images of Ki-67 protein expression in colon tissues, indicating epithelial proliferative activity (10 × magnification). **C** Representative immunofluorescence (IF) images of ZO-1 and claudin protein expression in colon tissues, reflecting intestinal tight junction integrity (20 × magnification)

Given that inflammatory infiltration and barrier disruption are pivotal in CAC pathogenesis [23, 24], we next examined the effect of TA on mucosal histopathology. H&E staining revealed that AOM/DSS induction glandular architecture disruption, massive inflammatory cell infiltration, and loss of epithelial integrity. Strikingly, TA treatment, particularly at the high dose, markedly attenuated these pathological changes (Fig. 2A and B). Tight junction proteins, such as ZO-1 and Claudin-1, are critical for maintaining intestinal barrier integrity. Immunofluorescence analysis revealed that AOM/DSS treatment disrupted junctional continuity and reduced the apical belt-like localization of both proteins, whereas TA treatment preserved tight junction integrity, consistent with the histopathological observations (Fig. 2C). Collectively, these data indicate that TA protects against CAC by restoring intestinal barrier function.

### TA suppresses pro-inflammatory cytokine release and neutrophil over- recruitment by downregulating of CXCL1/2 expression

**Fig. 3:**
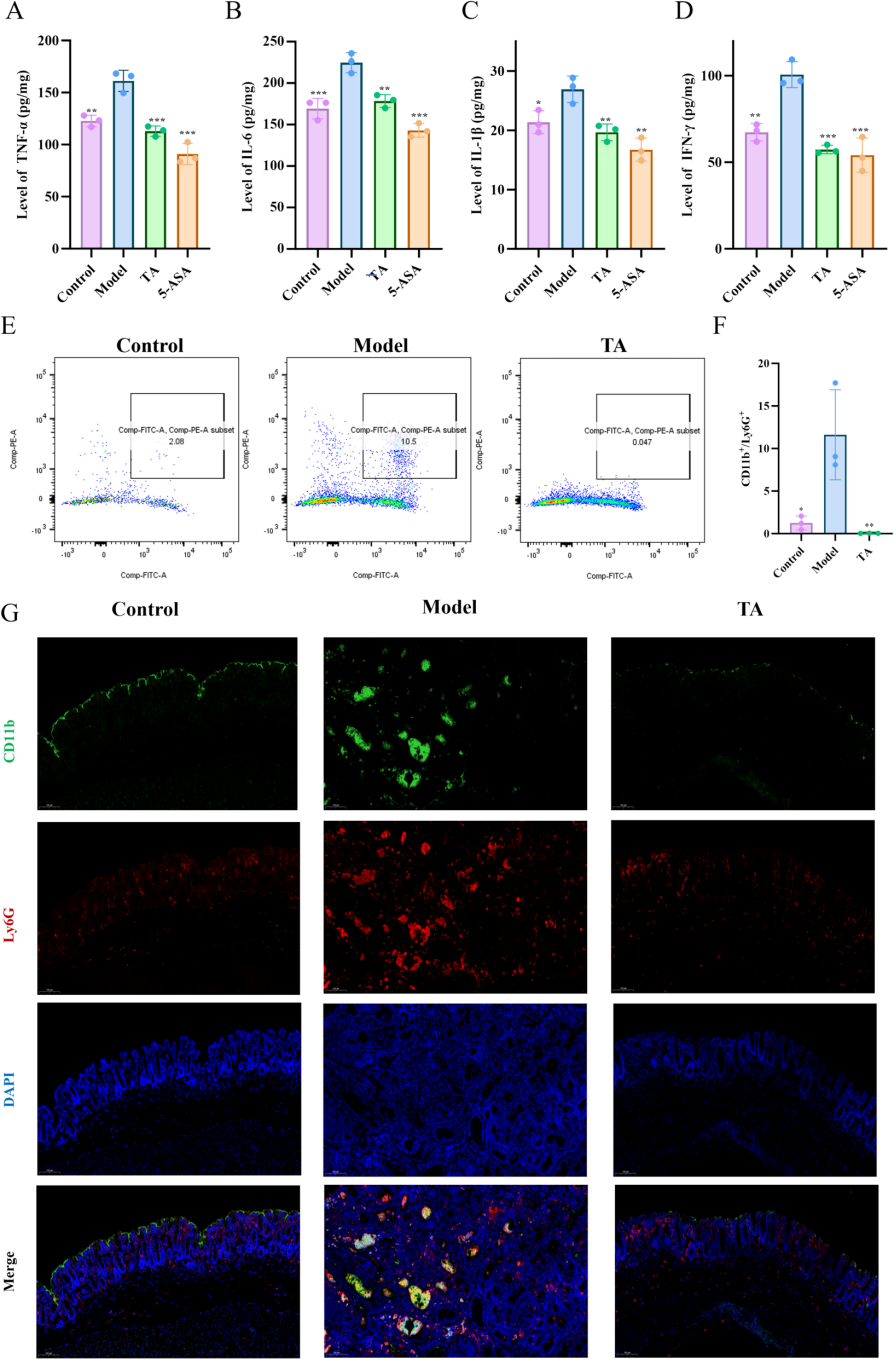
TA alleviates inflammatory responses and reduces excessive neutrophil recruitment in colon tissues. **A**–**D** Levels of inflammatory cytokines, including IL-1β, IL-6, IFN-γ, and TNF-α, in colon tissues from different treatment groups. **E**–**F** Quantitative analysis of neutrophil populations in colon tissues detected by flow cytometry. **G** Representative immunofluorescence (IF) images showing neutrophil infiltration in colon tissues. All data are expressed as mean ± SD. *P < 0.05, **P < 0.01, ***P < 0.001

The attenuation of mucosal damage was accompanied by a significant suppression of the colonic levels of key pro-inflammatory cytokines, including IL-6, TNF-α, IL-1β, and IFN-γ, in TA treated mice, to an extent comparable to the positive control 5-ASA (Fig. 3A-D). Neutrophils are key drivers of such inflammation-induced tumorigenesis [25, 26]. Given the observed suppression of cytokines and tissue inflammatory suppression, we hypothesized that TA acts by modulating neutrophil recruitment. Flow cytometry analysis confirmed a significant increase in neutrophil infiltration within the colon tissues of model mice, which was effectively suppressed by TA treatment (Fig. 3Eand 3F). This finding was further corroborated by immunofluorescence staining, which showed abundant Ly-6G⁺ neutrophils infiltrating the colonic lamina propria in model mice, an effect potently inhibited by TA treatment (Fig. 3G).

**Fig. 4:**
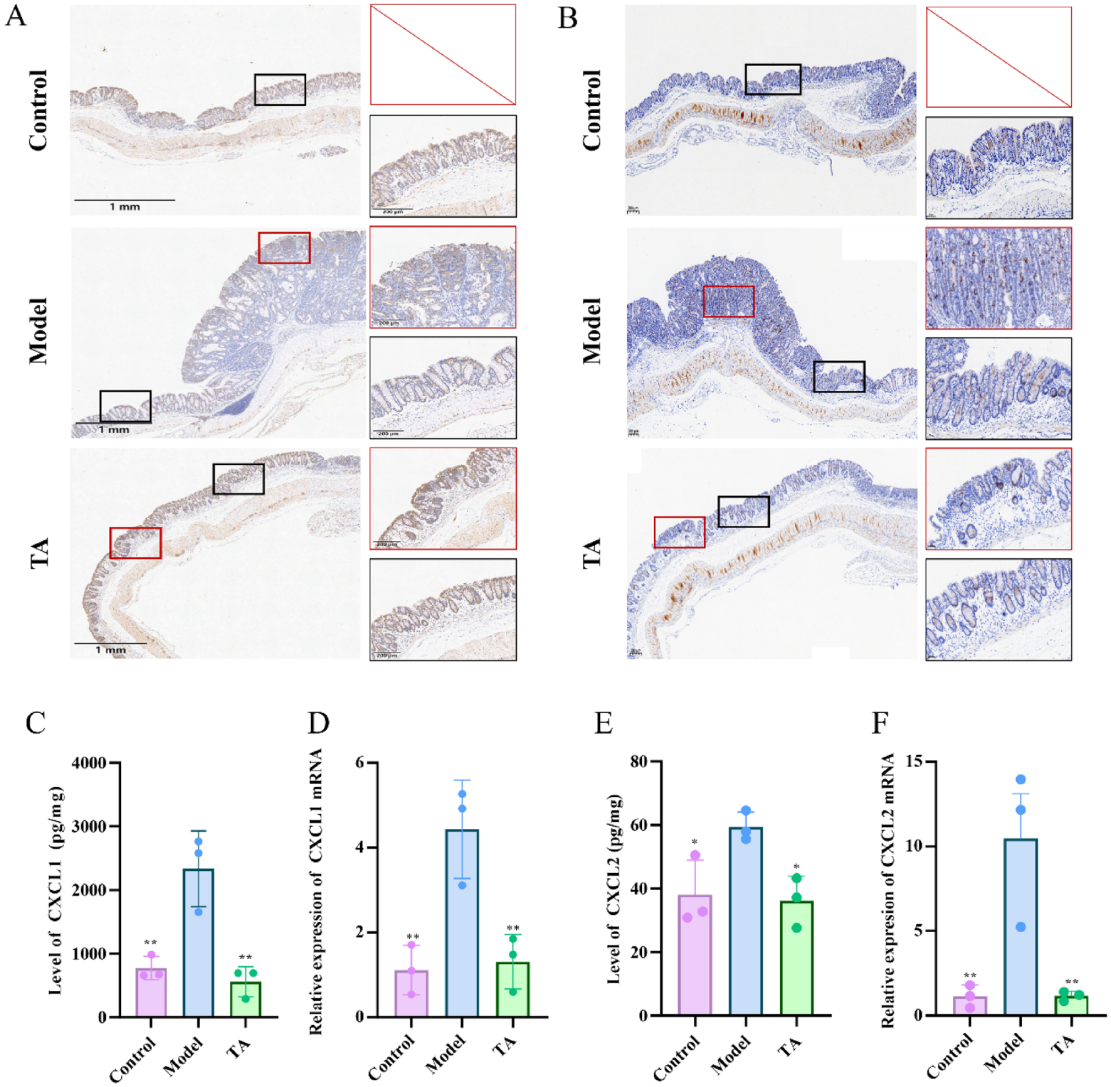
TA suppresses neutrophil over-recruitment by downregulating CXCL1 and CXCL2 expression in colon tissues. **A**, **B** Representative immunohistochemical (IHC) staining images of CXCL1 and CXCL2 protein expression in colon tissues (10 × magnification). **C**, **E** Protein levels of CXCL1 and CXCL2 in colon tissues measured by ELISA. **D**, **F** Relative mRNA expression levels of CXCL1 and CXCL2 measured by RT–PCR. All data are expressed as mean ± SD. *P < 0.05, **P < 0.01

As the CXCL1/2-CXCR2 axis is a primary chemoattractant pathway for neutrophils, we investigated whether TA affects this axis [27, 28]. Immunohistochemistry, ELISA, and RT-PCR analyses consistently showed that the protein and mRNA expression levels of both CXCL1 and CXCL2 were significantly upregulated in the colonic tissues of model mice. Importantly, TA treatment markedly downregulated their expression (Fig. 4A–F). These results suggest that the inhibition of neutrophil recruitment by TA is mediated, at least in part, through the suppression of the neutrophil chemoattractants CXCL1 and CXCL2.

### TA impedes neutrophil recruitment by interfering with the CXCL1/2-CXCR2 axis

**Fig. 5:**
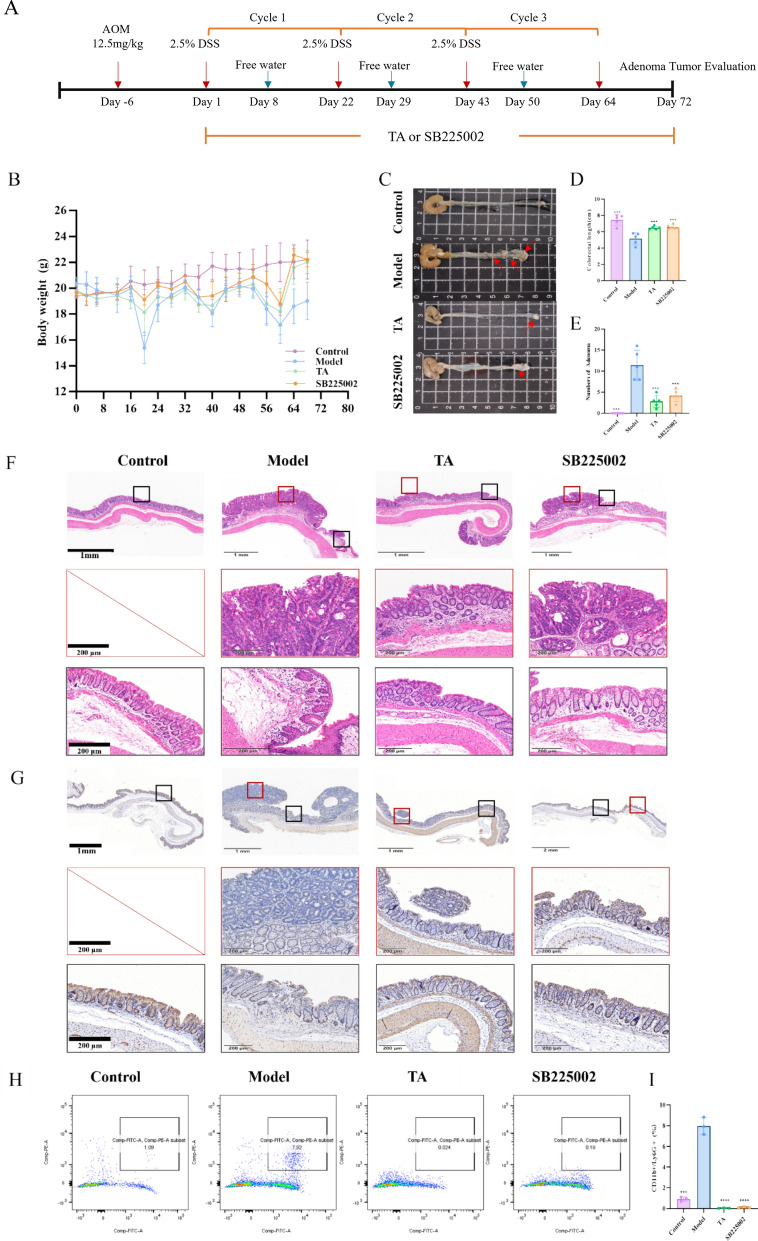
TA impedes neutrophil recruitment through modulation of the CXCL1/CXCL2–CXCR2 axis in vivo. **A** Experimental design of the in vivo model. **B** Body weight changes of mice in different treatment groups. **C**–**E** Colon length and adenoma number in each group, with representative macroscopic images and quantitative analysis. **F** Representative hematoxylin and eosin (**H**&**E**) staining images of colon tissues (10 × magnification). **G** Representative immunohistochemical (IHC) staining images of Ki-67 protein expression in colon tissues (10 × magnification). **H**–**I** Quantitative analysis of neutrophil populations in colon tissues detected by flow cytometry. All data are expressed as mean ± SD. *P < 0.05, **P < 0.01, ***P < 0.001, ****P < 0.0001.

To further confirm that TA acts through the CXCL1/2-CXCR2 axis, we utilized the specific CXCR2 antagonist SB225002 as an interventional control. Similar to the effects of TA, SB225002 treatment significantly ameliorated AOM/DSS-induced colon shortening and reduced adenoma formation (Fig. 5B–E and Supplementary Fig. 4). Histological analysis demonstrated that both TA and SB225002 markedly improved mucosal architecture and reduced inflammatory infiltration (Fig. 5F). Furthermore, immunohistochemical staining for the proliferation marker Ki-67 revealed that both treatments significantly suppressed abnormal proliferation of epithelial cells (Fig. 5G). Critically, Flow cytometry analysis confirmed that the attenuation of pathological changes by both TA and SB225002 was associated with a comparable reduction in neutrophil infiltration (Fig. 5H-I). These parallel outcomes strongly suggest that TA exerts its protective effects by disrupting the CXCL1/2-CXCR2 signaling axis, thereby limiting pathogenic neutrophil recruitment.

### TA suppresses the NOD/NF-κB pathway to downregulate CXCL1/2 expression

**Fig. 6:**
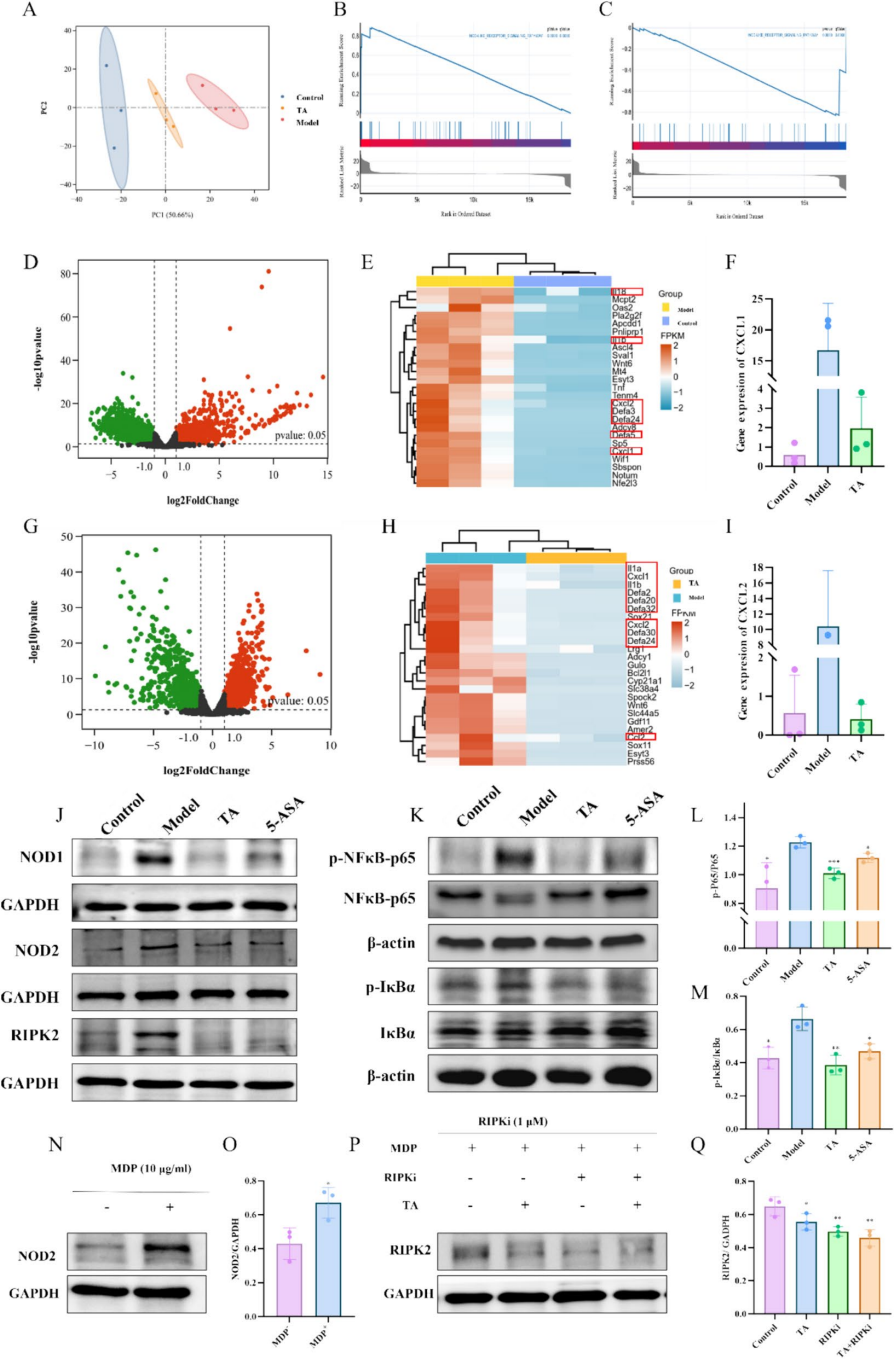
Transcriptomic profiling and signaling pathway analysis reveal the molecular mechanisms underlying TA-mediated suppression of neutrophil recruitment. **A** Principal component analysis (PCA) of RNA-seq data from intestinal tissues of AOM/DSS-treated mice with or without TA treatment. **B**, **C** Gene set enrichment analysis (GSEA) of differentially expressed genes derived from RNA-seq data of tumor intestinal tissues in AOM/DSS-induced mice with or without TA treatment (adjusted *P* < 0.05). **D**–**G** Volcano plots showing differentially expressed genes in intestinal tissues between the indicated groups. **E**–**H** Heatmaps of differentially expressed genes in intestinal tissues from each group. Differentially expressed genes were identified using the criteria of *P* < 0.05 and |log₂ fold change|> 1. **F**, **I** Relative mRNA expression levels of CXCL1 and CXCL2 in intestinal tissues from each group. **J**–**M** Western blot analysis of key proteins involved in the NOD/NF-κB signaling pathway in tumor intestinal tissues from mice under different treatment conditions, with quantitative densitometric analysis. **N**–**O** Western blot validation of NOD2 protein expression in Caco-2 cells, with quantitative densitometric analysis. **P**–**Q** Western blot validation of RIPK2 protein expression in Caco-2 cells, with quantitative densitometric analysis. Quantitative data from in vitro experiments are presented as mean ± SD from three independent experiments (n = 3). *P* < 0.05, *P < 0.01 versus the model group

To explore the signaling pathways potentially involved in TA-mediated regulation of CXCL1/2 expression, we performed RNA sequencing. Transcriptomic analysis identified numerous differentially expressed genes between the Model and TA groups (Fig. 6A). GSEA of these genes revealed a significant suppression of the NOD-like receptor signaling pathway in the TA group (Figs. 6 B, C and Supplementary Fig. 5A-5D). Further analysis confirmed that the expression of CXCL1 and CXCL2 was significantly downregulated by TA treatment (Fig. 6D–I).

To validate these findings, we examined the key proteins in the NOD/NF-κB pathway by Western blot. The results demonstrated that TA treatment significantly inhibited the expression of NOD1, NOD2, RIPK2, and the phosphorylation of IκBα and NF-κB p65 (Fig. 6J–M and supplementary figure 6 A-C). This indicates that TA suppresses the activation of the NOD/NF-κB pathway, a key regulator of CXCL1/2 transcription.

Given that NOD2 is a central mediator of intestinal inflammatory signaling and that muramyl dipeptide (MDP) is a specific agonist of NOD2, we next performed functional assays focusing on the NOD2–RIPK2 axis [29, 30]. MDP stimulation markedly upregulated NOD2 expression in Caco-2 cells, whereas treatment with TA or a RIPK2 inhibitor significantly suppressed RIPK2 expression. Notably, combined treatment with TA and the RIPK2 inhibitor exhibited a stronger inhibitory trend compared with RIPK2 inhibition alone, suggesting that the anti-inflammatory effects of TA may, at least in part, be mediated through modulation of the NOD2–RIPK2 signaling pathway. (Fig. 6N–Q).

### Molecular docking predicts strong binding of TA constituents to key NOD pathway proteins

**Fig. 7:**
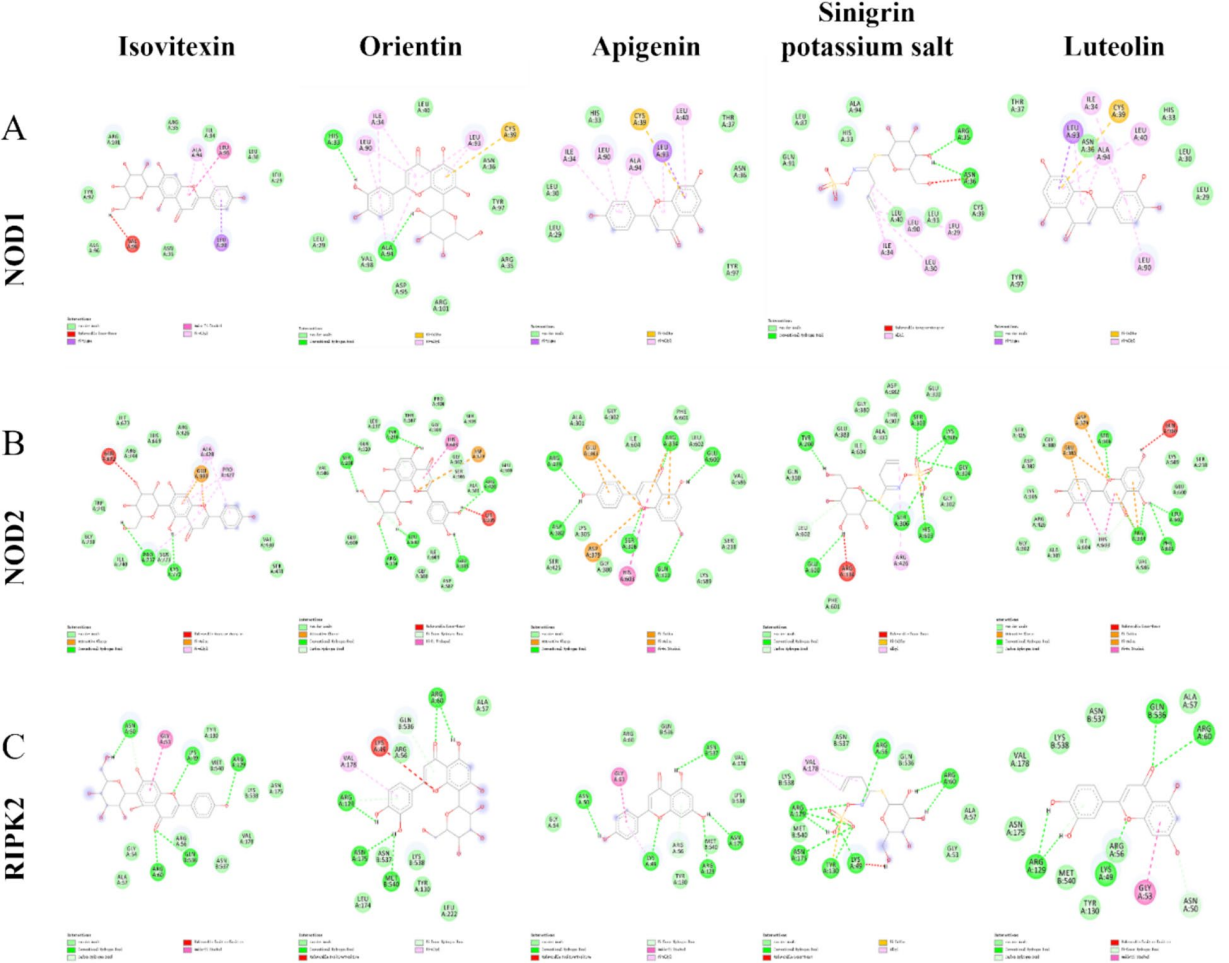
Molecular docking analysis of the major bioactive constituents of TA with core proteins of the NOD signaling pathway. **A**–**C** Representative molecular docking models showing the predicted binding of major TA constituents, including isovitexin, orientin, apigenin, sinigrin potassium salt and luteolin, with NOD1 (**A**), NOD2 (**B**), and RIPK2 (**C**), respectively. The docking results suggest favorable interactions between TA constituents and key proteins involved in NOD signaling

To explore the potential molecular interactions underlying TA's mechanism, we performed molecular docking analysis with its major bioactive components. The results indicated that the major bioactive components of TA, such as sinigrin, isovitexin, and orientin, were predicted to stably bind to the core proteins of the NOD pathway (NOD1, NOD2, and RIPK2) with favorable binding energies (ranging from −6.0 to −8.6 kcal/mol) (Fig. 7A–C, Table 1) [31, 32]. Notably, flavonoid compounds such as orientin, apigenin, luteolin, and isovitexin exhibited higher predicted binding affinities toward NOD2 than toward NOD1. In addition, luteolin showed particularly strong binding affinity to RIPK2, whereas sinigrin potassium salt displayed comparatively weaker interactions with the tested targets. Collectively, these findings suggest that luteolin may represent a key bioactive constituent contributing to the anti-inflammatory and anti-tumor effects of TA.

**Table 1:** Molecular docking scores of compounds with target proteins (kcal/mol)

	NOD1	NOD2	RIPK2
Isovitexin	−7	−8.3	−6.1
Orientin	−7.2	−8.7	−7
Apigenin	−7.3	−8.6	−6.9
Sinigrin potassium salt	−5.3	−7.7	−6.3
Luteolin	−7.3	−8.6	−7.6

## Discussion

In this study, we provide compelling evidence that TA may confer protection against CAC in a murine model. Our findings delineate a coherent mechanistic pathway whereby TA attenuates neutrophil-driven pathology by suppressing the NOD/NF-κB signaling axis, leading to the downregulation of the key neutrophil chemoattractants CXCL1 and CXCL2, and potentially preserving intestinal barrier integrity. This multi-faceted action highlights the potential of TA as a novel therapeutic strategy for intercepting the inflammation-to-cancer sequence in the colon.

We first established that TA effectively alleviated the hallmark features of AOM/DSS-induced CAC, including improving survival, reducing colon shortening, and suppressing tumor burden. rucially, histological analysis revealed that TA treatment markedly ameliorated mucosal damage and inflammatory infiltration. A key pathogenic event in CAC is the compromise of the intestinal barrier, which facilitates the translocation of microbial products and perpetuates inflammation [16, 33]. The resulting repeated injury and regeneration can promote epithelial dysplasia and carcinogenesis [15]. Our data demonstrate that TA may play a critical role in maintaining barrier function, as evidenced by the restoration of the tight junction proteins ZO-1 and Claudin-1. This preservation of the physical barrier could represent a fundamental step in the mechanism of TA, by limiting the exposure of the mucosal immune system to pro-tumorigenic stimuli.

Given the well-established central role of neutrophils in driving the progression from chronic inflammation to cancer [34, 35], we focused on whether TA modulates their recruitment. Our results show that TA treatment may reduced neutrophil infiltration into colonic tissues. This reduction was associated with a marked decrease in the levels of pivotal pro-inflammatory cytokines, including IL-6, TNF-α, IL-1β, and IFN-γ, which are known to establish a pro-tumorigenic microenvironment. More specifically, the CXCL1/2-CXCR2 axis appears to be a target of TA. The concordant downregulation of CXCL1 and CXCL2 at both the mRNA and protein levels by TA, coupled with the fact that a specific CXCR2 antagonist (SB225002) phenocopied the protective effects of TA on neutrophil recruitment, tumorigenesis, and epithelial proliferation, is consistent with the hypothesis that TA may acts through this specific chemokine axis to curb pathogenic neutrophil influx.

To elucidate the upstream signaling events, we employed an unbiased transcriptomic approach. GSEA pointed to the NOD-like receptor signaling pathway as being significantly suppressed by TA. This finding is highly relevant, as previous studies have shown that persistent microbial stimulation through pattern recognition receptors such as NOD1 and NOD2 is closely associated with inflammation-driven carcinogenesis in IBD [18, 36]. In particular, our prior work demonstrated that TA treatment could significantly reshape the gut microbiota in a colitis model [18]. Building upon that finding, the current study aimed to investigate the consequent downstream host signaling. Upon ligand recognition, NOD1 and NOD2 recruit RIPK2, leading to the activation of the IKK complex and the canonical NF-κB pathway. Sustained NF-κB activation then has been reported to promote the transcription of pro-inflammatory genes, including CXCL1 and CXCL2 [37, 38]. Our validation experiments showed that TA treatment was associated with decreased protein levels of NOD1, NOD2, RIPK2, and reduced phosphorylation of IκBα and NF-κB p65. These observations suggest that TA could interfere with this signaling cascade, potentially limiting the NOD/NF-κB-dependent induction of neutrophil chemoattractants. In addition, In Caco-2 cells, treatment with TA or a RIPK2 inhibitor significantly attenuated MDP-induced activation of the NOD2 signaling pathway. Importantly, combined treatment did further enhance inhibition compared with RIPK2 inhibition alone. These findings are indicating that TA exerts its anti-inflammatory effects, at least in part, through modulation of the NOD2–RIPK2 pathway.

Molecular docking analysis provides a structural basis for the inhibitory effects of TA on the NOD signaling pathway, suggesting that its bioactive constituents may directly interact with key proteins, including NOD1, NOD2, and RIPK2. These interactions exhibited favorable binding energies and stable conformations, supporting the multi-target regulatory potential of TA. Notably, several compounds present in TA, including hesperidin, apigenin, luteolin, and isovitexin, showed higher predicted binding affinities toward NOD2 than toward NOD1, indicating a preferential modulation of the NOD2-centered inflammatory axis. Among these compounds, luteolin displayed relatively strong binding affinity to RIPK2, a critical adaptor kinase linking NOD2 activation to NF-κB signaling. Consistent with this, the docking results provide a mechanistic explanation for our functional findings that TA suppresses NOD/NF-κB activation and downstream CXCL1/2 expression. Given that TA is a multi-component herbal preparation, its pharmacological effects are likely mediated by synergistic actions of multiple constituents rather than a single compound. Although luteolin may represent an important contributor to the anti-inflammatory and anti-tumor effects of TA, the relative roles of individual components warrant further investigation through component fractionation and targeted functional validation.

Collectively, our results suggest a previously uncharacterized mechanism whereby TA may ameliorate CAC, possibly by modulating the NOD/NF-κB/CXCL1/2 axis to dampen neutrophil recruitment. This action could contribute to limiting the cycle of inflammation, barrier dysfunction, and tumorigenesis. Given the limitations of current IBD therapies [8], these findings position TA as a promising candidate worthy of further investigation as a potential preventive or adjunctive therapy for CAC. To fully elucidate its therapeutic potential, future research should aim to identify the primary active compounds in TA, validate the causal role of the NOD pathway through genetic or cell‑specific models, and systematically examine how TA‑modulated gut microbiota contribute to host signaling and cancer prevention. Together, these efforts will advance a more integrated understanding of the mechanism of action of TA against CAC.

## Conclusion

In summary, this study provides evidence that TA may attenuate the development of AOM/DSS-induced CAC in mice. Its protective effects could involve suppression of the NOD/NF-κB signaling pathway, potentially resulting in reduced expression of the neutrophil chemoattractants CXCL1/CXCL2, inhibition of excessive neutrophil infiltration, and preservation of intestinal barrier integrity. These findings suggest the therapeutic potential of TA as a novel natural agent for the prevention and treatment of CAC, while further studies are warranted to confirm the underlying mechanisms.

## Supplementary Information

Supplementary file 1.

## Data Availability

The data that support the findings of this study are available from the corresponding author upon reasonable request.

## Abbreviations

5-ASA: 5-Aminosalicylic acid
AOM: Azoxymethane
CRC: Colorectal cancer
CAC: Colitis associated colorectal cancer
Cxcl1: C-X-C Motif Chemokine Ligand 1
Cxcl2: C-X-C Motif Chemokine Ligand 2
CXCR2: C-X-C motif chemokine receptor 2
DSS: Dextran sulfate sodium
DMEM: Dulbecco's modified Eagle's medium
ELISA: Enzyme-linked immunosorbent assay
FBS: Fetal bovine serum
GSEA: Gene set enrichment analysis
H&E: Hematoxylin and eosin
IHC: Immunohistochemical
IF: Immunofluorescence
IBD: Inflammatory bowel disease
IL-1β: Interleukin-1 beta
IL-6: Interleukin-6
Ki-67: Marker of proliferation Ki-67
MIP-2/CXCL2: Mouse macrophage inflammatory protein-2
MDP: Muramyl dipeptide
NOD1: Nucleotide Binding Oligomerization Domain 1
NOD2: Nucleotide Binding Oligomerization Domain 2
NF-κB p65: Nuclear factor kappa-B p65
Phospho- NF-κB p65: Phospho-Nuclear factor kappa-B p65
PCA: Principal component analysis
RT-qPCR: RNA extraction and quantitative real-time PCR
RIPK2: Receptor-interacting serine/threonine-protein kinase 2
RIPKi: RIPK2 kinase inhibitor
SB225002: CXCR2 antagonist
TNF-α: Tumor Necrosis Factor alpha
TA: Thlaspi arvense
YYFZBJS: Yiyi Fuzi Baijiang San
ZO-1: Zonula Occludens-1
