# Supplementary material for: Thlaspi arvense attenuates colitis-associated colorectal tumorigenesis through suppression of neutrophil recruitment via the NOD/NF-κB pathway
Source: Chin Med. 2026 Apr 17;21:119. doi: 10.1186/s13020-026-01381-5 (PMC13088638; doi:10.1186/s13020-026-01381-5)
Supplement: Supplementary file 1 [file 13020_2026_1381_MOESM1_ESM.docx]

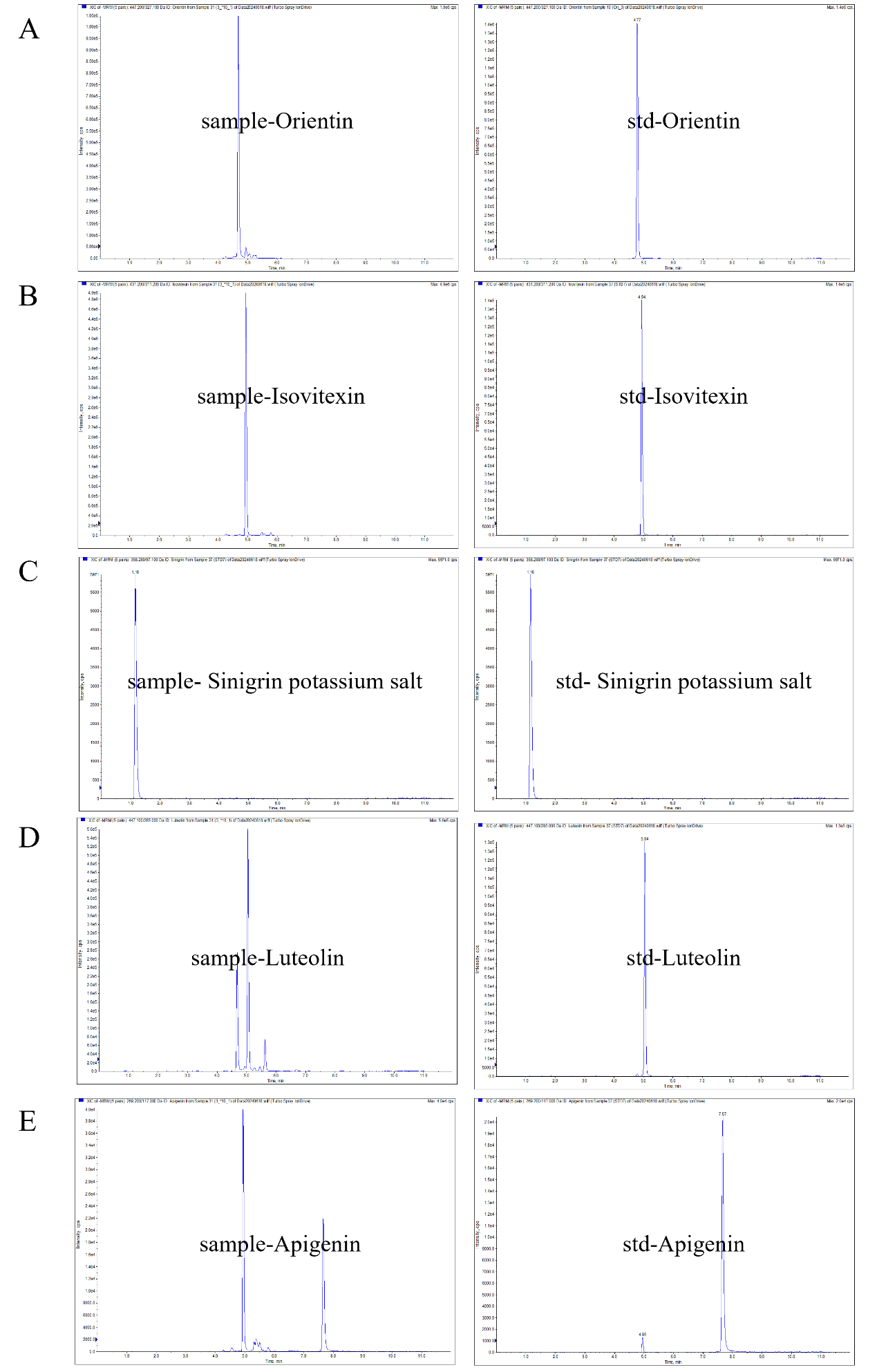


**Supplementary Figure 1** The quality control and analytical chromatograms of the main active ingredients of *Thlaspi arvense*.

**Supplementary Table 1 Standard curves and content information of key components of *Thlaspi arvense*.**

| **Compound** | **The standard curve** | **Linear range(ng/mL)** | **R** | **Sample(ng/mg)^*^** |
| --- | --- | --- | --- | --- |
| Apigenin | y=1670x+5360 | 25-4000 | 0.9996 | 84.6 |
| Sinigrin potassium salt | y=454x−533 | 30-4800 | 0.9999 | 2866.0 |
| Isovitexin | y=5050x−20300 | 37.5-6000 | 0.9999 | 4509.3 |
| Oritentin | y=1280x−15000 | 25-4000 | 0.9995 | 4151.1 |
| Luteolin | y=8730x+19000 | 22.5-3600 | 1 | 311.5 |

***** Content concentration of freeze-dried powder.

**
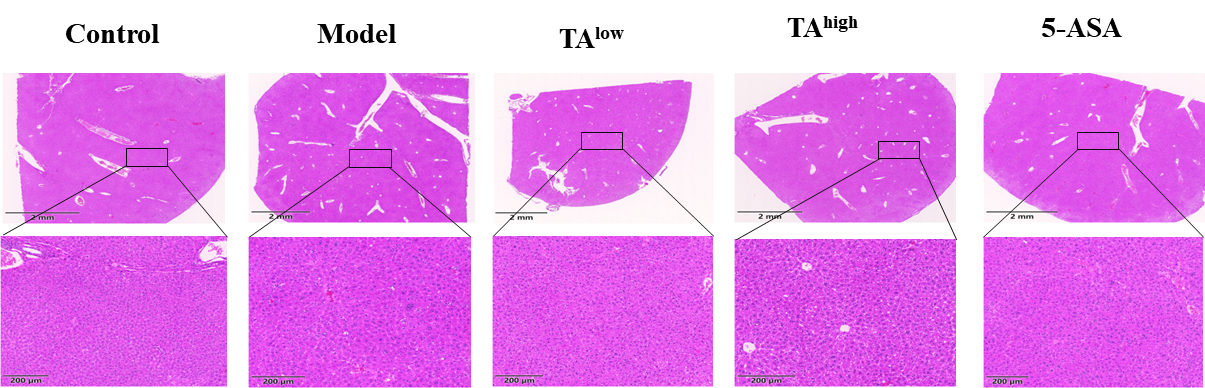
**

**Supplementary Figure 2** Representative Liver images of mice.


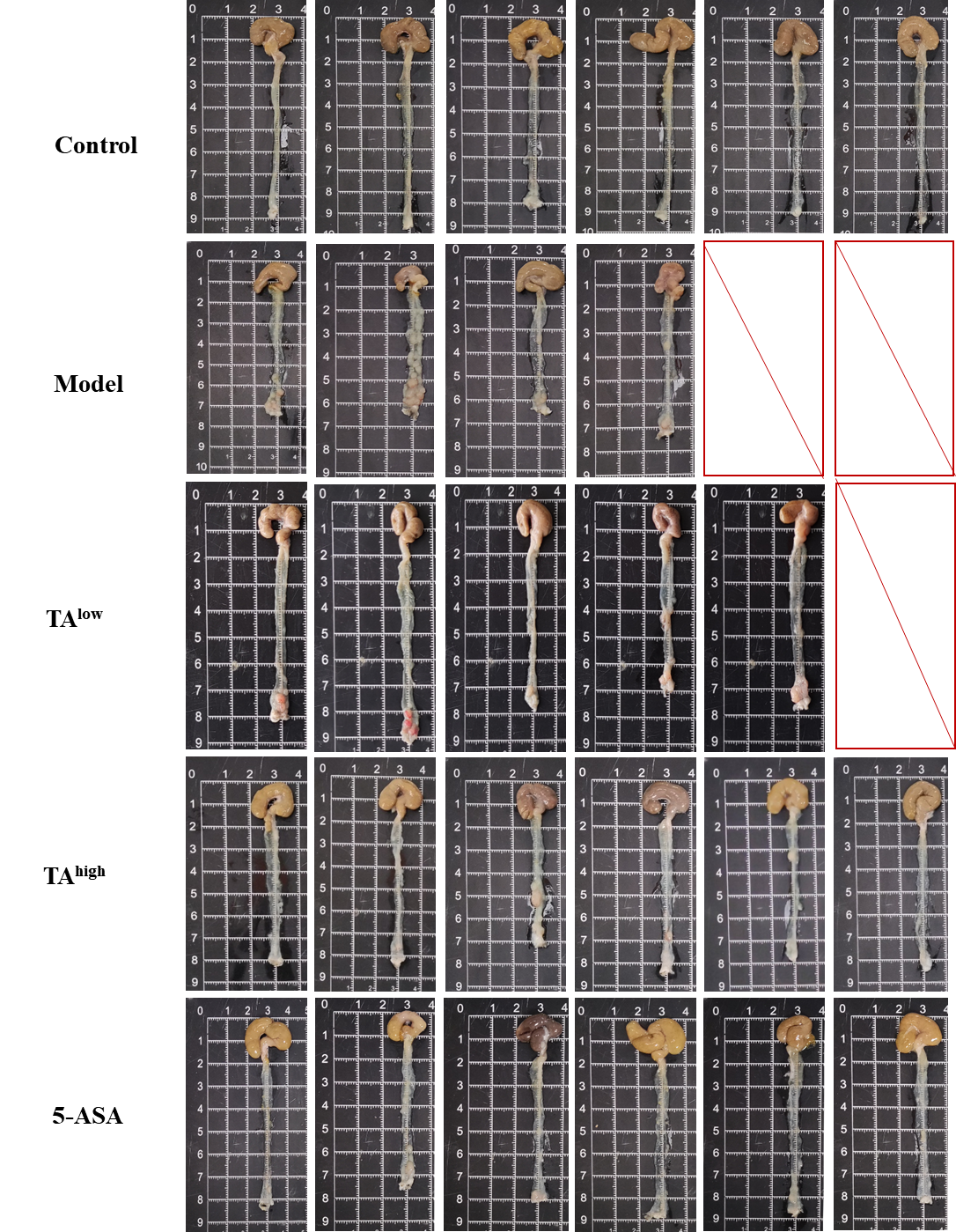


**Supplementary Figure 3** Mouse colorectal tissue images

**
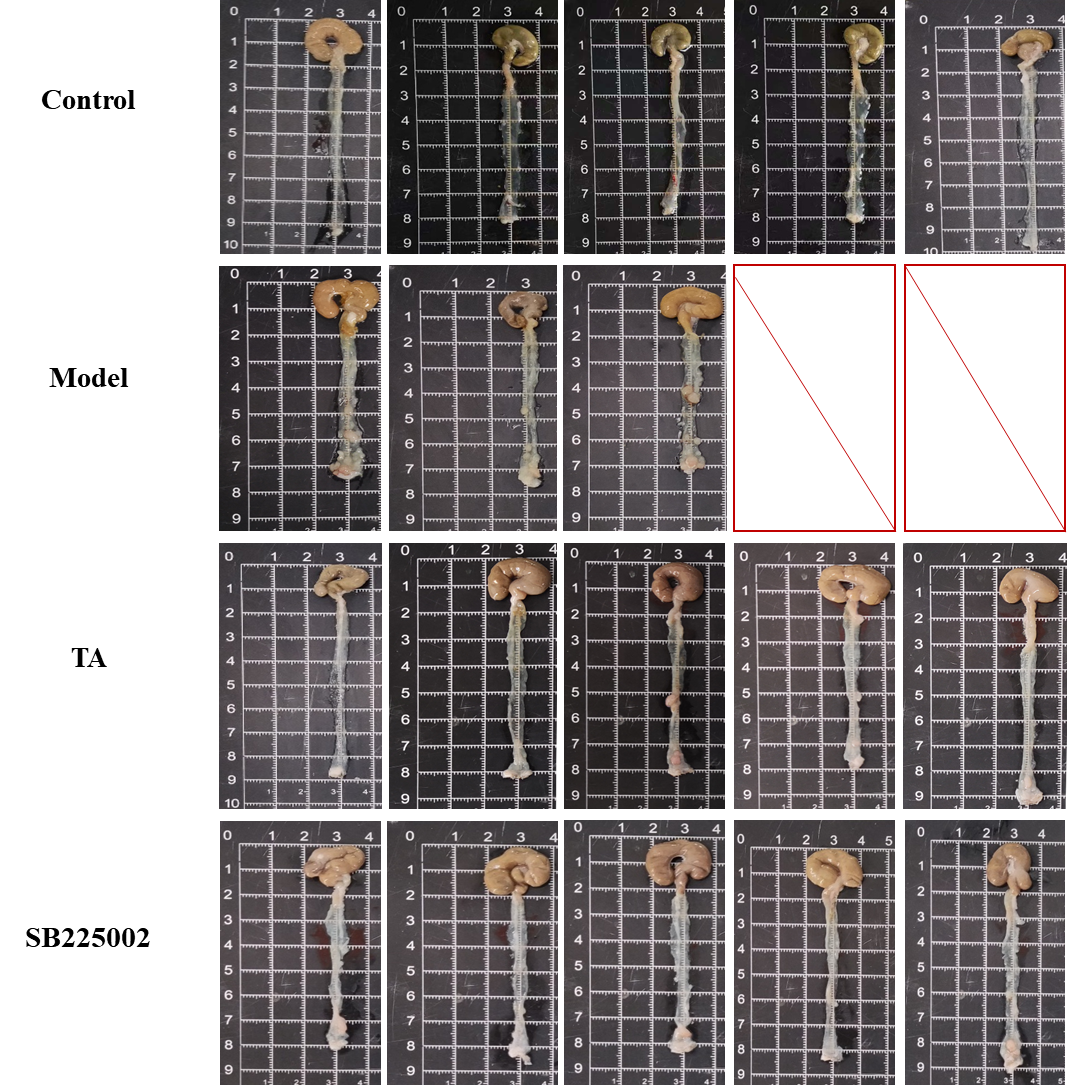
**

**Supplementary Figure 4** Mouse colorectal tissue images


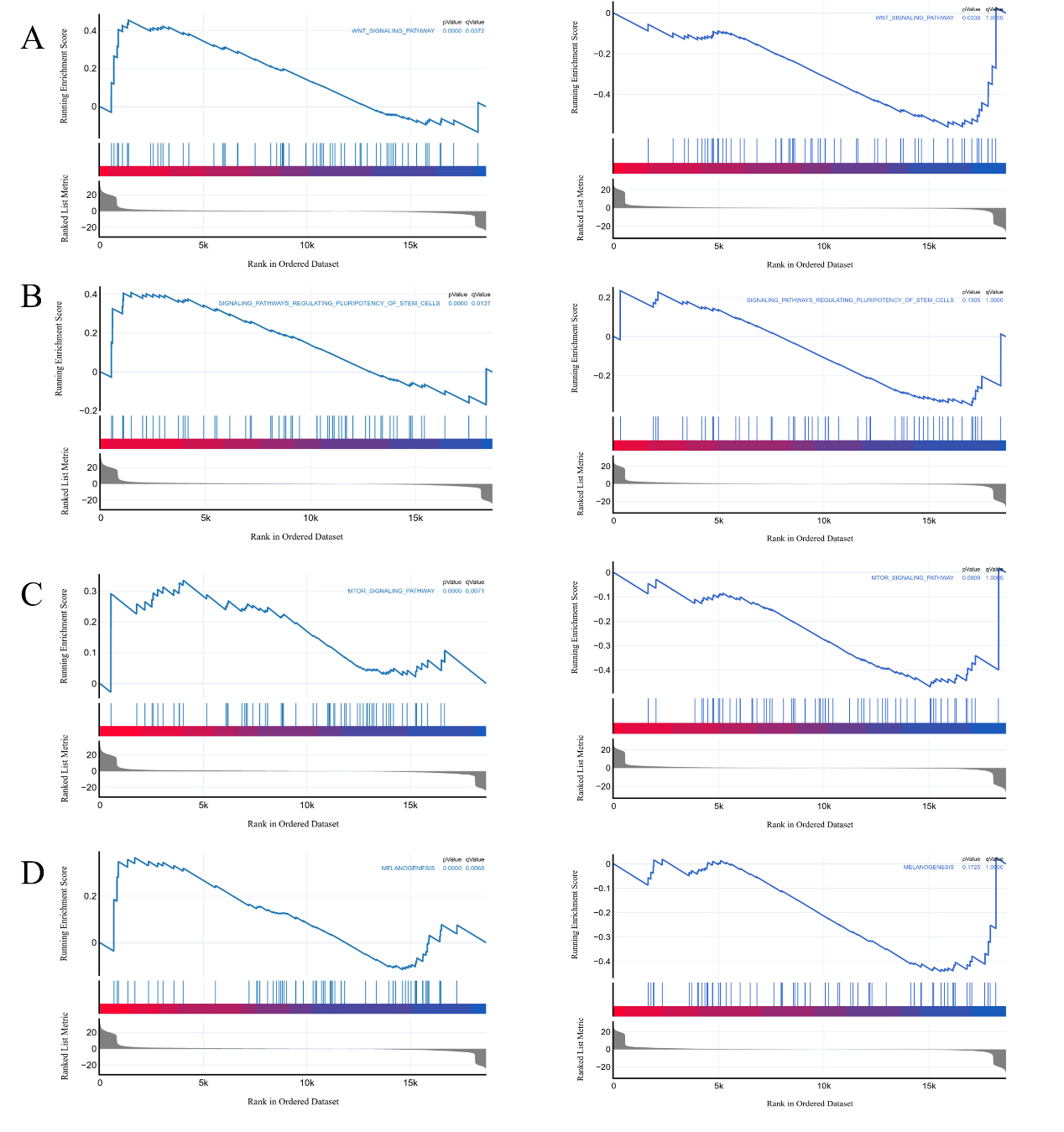


**Supplementary Figure 5** GSEA enrichment analysis of differentially regulated genes from RNA-seq data of tumor tissues in AOM/DSS-induced mice. Left: Mice without TA treatment; Right Mice with TA treatment.

**
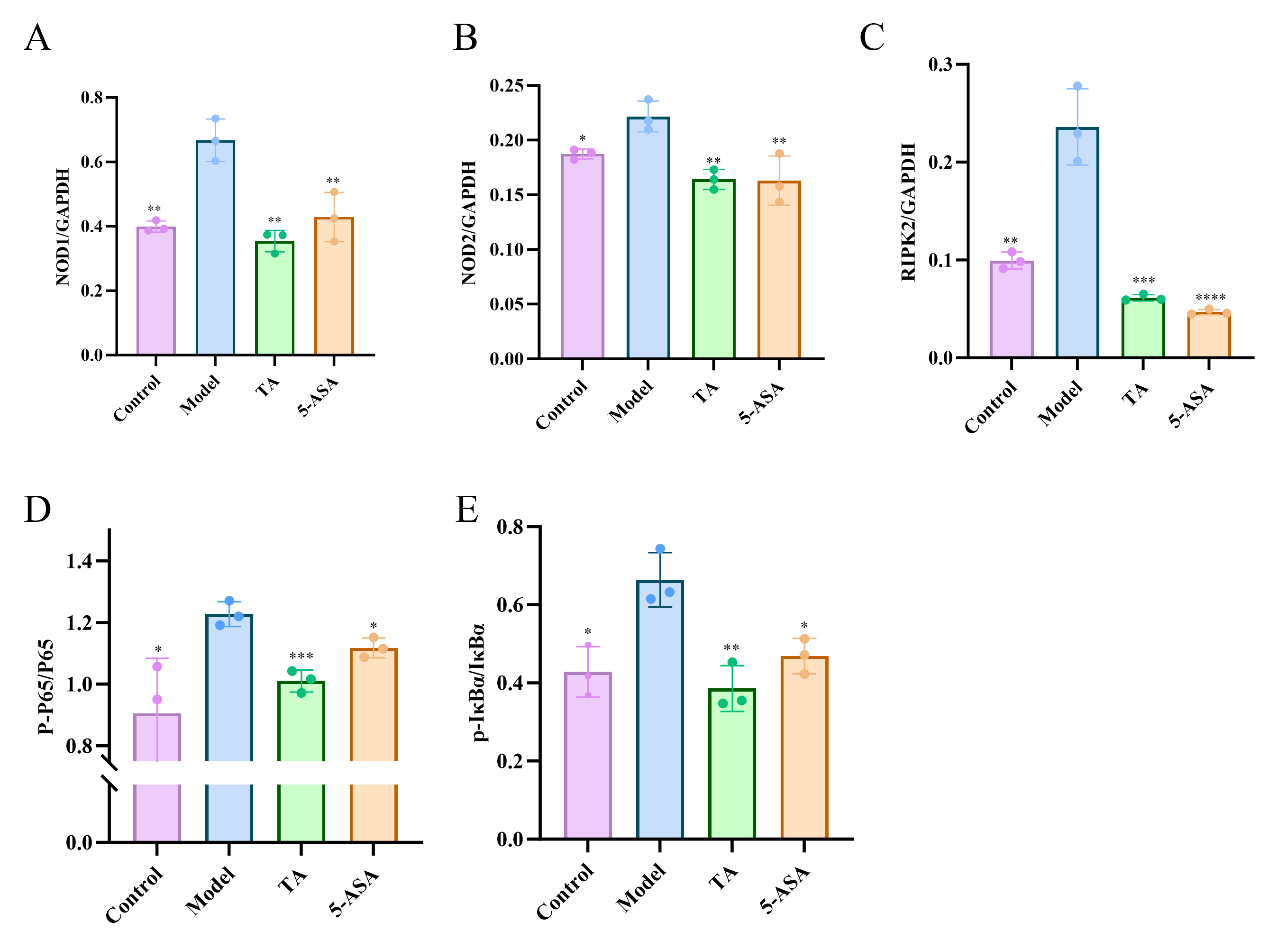
**

**Supplementary Figure 6** Western blot protein bands were quantitatively analyzed by grayscale using Image software. Data are presented as mean ± standard deviation from three independent experiments (n = 3). *P < 0.05, **P < 0.01, compared to the model group.

**
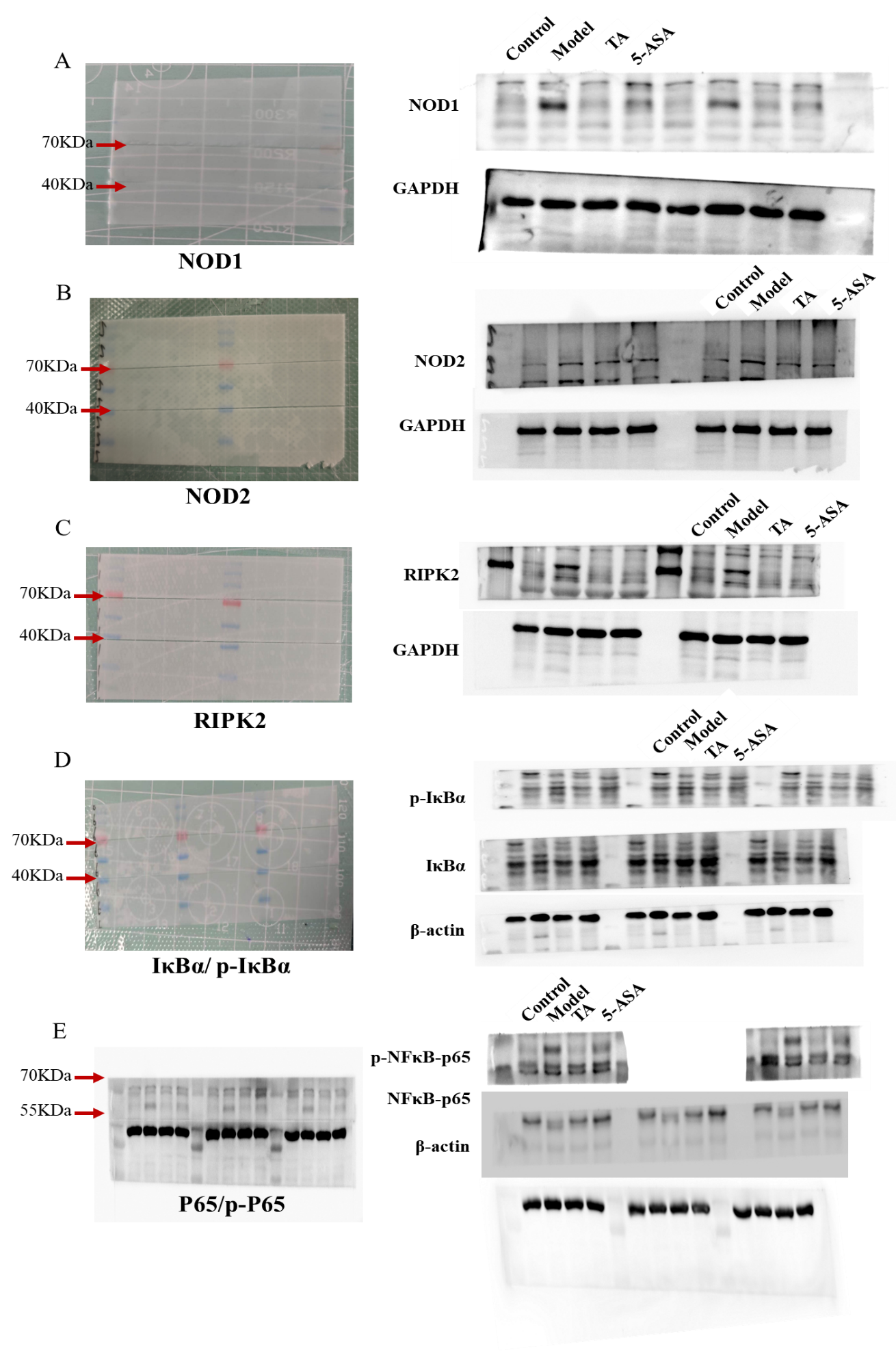
**

**Supplementary Figure 7 Uncropped blot for NOD1 (A), NOD2 (B), RIPK2 (C), p-IκBα/IκBα (D) and p-NFκB-p65/NFκB-p65 (E)**

**
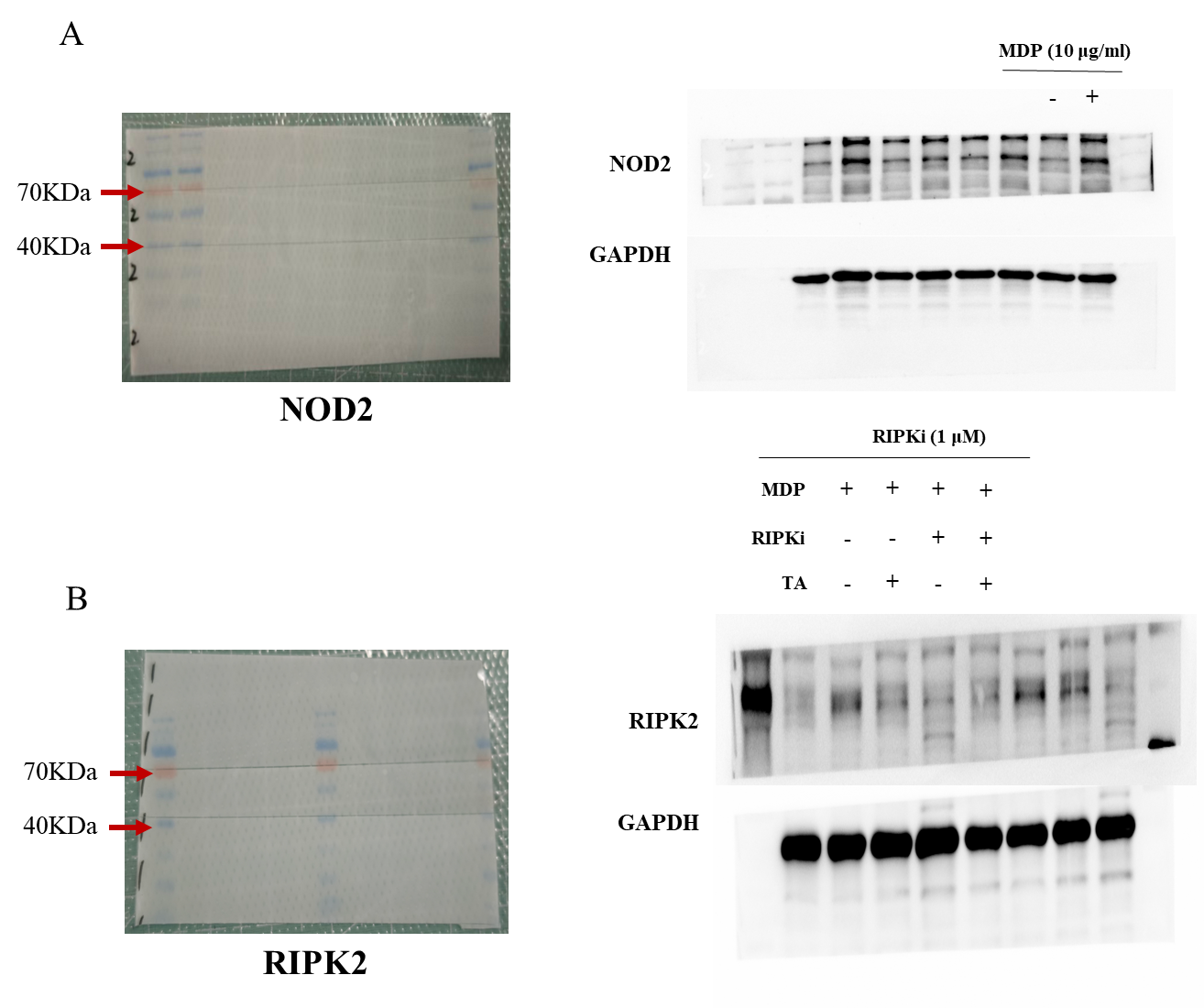
**

**Supplementary Figure 8 Uncropped blot for NOD2 (A) and RIPK2 (B)**
